# Selective Adsorption of Organic Micro-Pollutants by Smectite Clays Revealed from Atomistic Simulations

**DOI:** 10.3390/ijms241914781

**Published:** 2023-09-30

**Authors:** Mathieu Cancade, Thomas Thiebault, Pierre Mignon

**Affiliations:** 1 Institut Lumière Matière, UMR 5306, Université Claude Bernard Lyon 1, CNRS, Université de Lyon, 69622 Villeurbanne, France; mathieu.cancade@ens-lyon.fr; 2 Milieux Environnementaux, Transferts et Interactions dans les Hydrosystèmes et les Sols, Sorbonne Université, CNRS, EPHE, PSL University, UMR 7619, 75005 Paris, France; thomas.thiebault@upmc.fr

**Keywords:** organic pollutants, adsorption, clay, molecular simulations, DFT

## Abstract

In this study, atomistic simulations were carried out to study the difference in the adsorption process between two similar molecules, diazepam and oxazepam, on Na^+^-montmorillonite. Kinetic and XRD measurements showed a contrasting adsorption mechanism of these two molecules, differing only by the presence/absence of methyl and hydroxyl groups, with a larger adsorption amount and intercalation for the oxazepam. The structural characterization of these molecules was investigated through DFT calculations and showed the vicinity of hydroxyl and carbonyl groups for only the chair conformation of oxazepam compared to the boat conformation. Classical molecular dynamics simulations of diazepam and the two forms of oxazepam on the external surface of Na^+^-montmorillonite highlighted the better coordination of the oxazepam-chair conformation, compared to its boat counterpart and diazepam. This has been confirmed through DFT calculations, from which a coordination energy that is greater by 10 kcal·mol^−1^ is observed. This strongly suggests that the experimentally observed intercalation of oxazepam occurs only in the chair form because of the strong coordination with the Na^+^ cation present in the Na-Mt interlayer. Classical MD simulations of the intercalated oxazepam chair molecule in the Na-Mt interlayer allowed the evaluation of the interlayer spacing d001, which was in very good agreement with the experimental XRD measurement.

## 1. Introduction

The fate of organic micro-pollutants (OMPs) in the environment is of major concern in society. After their use in everyday life, many organic compounds are released and can be detected in different compartments of the environment [1,2,3]. The occurrence of these OMPs (pesticides, industrial chemicals, pharmaceuticals, veterinary, steroids, personal care products, etc.) is regularly monitored in surface and ground waters, and, year after year, an increase in the amount and diversity of these substances is observed [1,4,5,6]. The way these compounds enter the environment depends on their use. They may be directly released into the natural environment (veterinary pharmaceuticals and pesticides) or pass through wastewater treatment plants (pharmaceuticals, industrial chemicals, etc.), via processes in which their removal remains incomplete [7,8,9,10,11]. In any case, their interactions with finely divided minerals will control, to a large extent, the fate of these organic substances in the natural environment and in engineered filtration beds. It is, then, essential to understand how organic substances interact with the minerals with which they come in contact after use. Among reactive minerals, clays are (i) naturally abundant environmental solids found in soils and sediments, (ii) offer significant surface area and ion exchange capacities, and (iii) are used as a low-cost material that might be used for pollutant removal in water treatment plants [12,13]. Smectite clays are layered (porous) silicates with the ability to swell, thus increasing their adsorption and water/cation exchange capacity, and, as such, they may actively participate in the retention and degradation of OMPs. In an effort to model and predict the fate of OMPs, it is crucial to assess and control their capacity to adsorb and generate/catalyze chemical reactions. Therefore, understanding the molecular interactions responsible for their retention is of prime interest.

OMP adsorption on clays has been studied experimentally, showing variability of adsorption strength depending on various conditions, including pH, ionic strength, and types of exchangeable cation [13,14,15,16,17]. Various techniques may be used to draw a scheme of the preponderant interactions driving the adsorption processes. Kinetic experiments and adsorption isotherms provide a general view and allow the calculation of numerous thermodynamic parameters, allowing the monitoring of the adsorption process according to two important variables, time and starting concentrations [13,15,16,17]; X-ray diffraction informs on the basal spacing between clay layers and allows researchers to determine if the adsorption occurs in the interlayer space or on external surfaces/edges [18,19]. As a complementary approach, theoretical calculations are often carried out, allowing researchers to evaluate the precise strength of intermolecular interactions involved in the adsorption process for various OMPs adsorbed on clay surfaces. From DFT static calculations, accurate energetics can be evaluated, for example, for atrazine (pesticide) on pyrophyllite and Ca^2+^-montmorillonite basal surfaces, which show strong adsorption on the substituted mineral surface because of strong coordination with the cation [20]. Similarly, metamitron (herbicide) and fenhexamid (fungicide) were found to adsorb fairly strongly on the same mineral because of unexpected cation complexation through the carbonyl oxygens [21]. The interaction adsorption energies of hexachlorobenzene on M^+/2+^-montmorillonite were found to correlate well with solid-liquid adsorption constants [22]. The molecular dynamic (MD) DFT calculation of atrazine in the Na^+^-montmorillonite/-beidellite minerals allowed previous researchers to highlight important intermolecular interactions [23]. The computed vibrational spectra allowed the assignment of the bands of the FTIR experimental spectra, enabling an estimation of the adsorbed amount of atrazine. Quantum chemical calculations provide very accurate data but are very demanding in terms of resources, restricting analysis to the use of models limited in size and duration. Classical MD studies based on the use of parametrized force fields, although less accurate, allow researchers to carry out investigations using extended models. Recent works showed that humic substances form aggregates on kaolinite surfaces stabilized through electrostatic interactions [24]. When confined in kaolinite nanopores, these aggregates may be fragmented in smaller species, which then adsorb quite strongly to the aluminol surface. A study of glyphosate on a kaolinite basal surface, investigated in classical MD simulations, shows that adsorption involves H-bonding interactions with the surface [25]. The adsorption free energy, evaluated using an umbrella sampling methodology, was measured for the neutral and anionic forms of glyphosate as −5 and −14 kJ.mol^−1^, respectively, thus showing the stabilizing electrostatic interaction of the charged form. Recently, we have shown that paracetamol and carbamazepine adsorb on a saponite surface stabilized from van der Waals interactions and also, for the latter molecule, from cation coordination interactions, providing greater adsorption stabilization. This was confirmed by the adsorption free energies evaluated from umbrella sampling simulations, thereby corroborating the kinetic and X-ray diffraction experiments [26].

**Figure 1:**
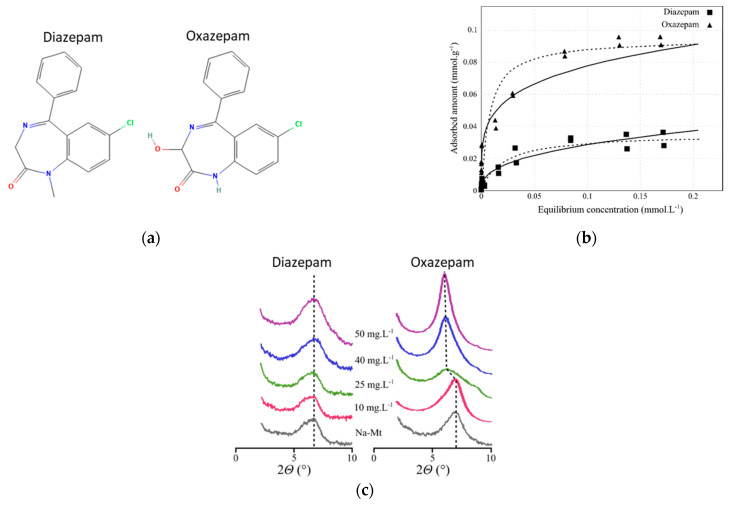
Experimental data taken from Ref. [16] and the chemical structures for diazepam and oxazepam: (**a**) chemical structures; (**b**) adsorption isotherms (black lines represent the Freundlich fits and dashed lines represent the Langmuir fits). (**c**) X-ray diffraction patterns of the OMPs-Na-Mt layered composite as a function of the starting OMP concentration. Figures (**b**) and (**c**) have been reproduced with *Elsevier/Colloids and Interface Science Communications* journal authorization.

In the present study, we investigated the adsorption process of two OMPs with very similar chemical structures, for which different and unexplained experimental adsorption data were observed [16]. The diazepam (DIA) and oxazepam (OXA) molecules are psychoactive drugs that are among the most frequently observed pharmaceutical compounds found in environmental compartments, due to their incomplete removal in wastewater treatment plants [27,28]. Their chemical structures only differ via the presence of a methyl group bound to the nitrogen heteroatom of the seven-membered cycle for diazepam, while a hydroxyl group is in the alpha position with respect to the carbonyl for oxazepam (Figure 1a). The adsorption mechanism on a Na^+^-exchanged montmorillonite clay (Na-Mt) was investigated via batch sorption isotherms and X-ray experiments at near-neutral pH (pH = 7.5) in an aqueous solution, i.e., the molecules are present in their neutral form. From the isotherms (Figure 1b), it is clear that moderate adsorption capacity was observed, showing rapid saturation of the adsorption sites of Na-Mt, with relative and non-negligible better adsorption for OXA compared to DIA. From the diffractograms (Figure 1c), it appears that the evolution of the basal spacing of Na-Mt with an increasing starting concentration of DIA did not show any shift. Conversely, the basal spacing of Na-Mt after interaction with OXA increased for the highest starting concentrations, from 10.4 Å to 13.3 Å. Thus, DIA adsorption was explained as occurring through weak interactions since it is neutral and does not intercalate, while for OXA, also in its neutral form, the observed adsorption and intercalation may involve a different mechanism. Two subsequent experimental studies analyzed the removal percentage of DIA and OXA in wastewater effluents (starting concentration of ~100 ng.L), showing limited removal capacity by using raw and Na-Mt [29,30]. However, the different adsorption mechanisms for these two molecules, despite small changes in their chemical structures, remained unexplained. For this reason, we investigated atomistic simulations in order to understand the molecular interactions that may be responsible for the distinct adsorption mechanisms for DIA and OXA. For this purpose, we performed classical MD simulations of the molecules interacting with the hydrated external surface of a Na-Mt model, analyzing the behaviors of the two molecules with the surface and Na^+^ cations. These results were then completed with high-level DFT calculations in order to confirm the observed differences in the intermolecular interactions in which DIA and OXA molecules are involved. It should be noted that atomistic calculations have already been used to investigate the adsorption modes of DIA on kaolinite [31] and vermiculite [32] surfaces, showing stabilizing H-bonding and cation bridging adsorption conformations, respectively. However, no in-depth study has yet investigated the difference between the adsorption mechanism of DIA and OXA and of similar molecules.

## 2. Results and Discussion

Several computational chemistry techniques have been used in this study, selected according to the desired properties and description. Generally, classical MD simulations allow the description of the dynamic properties of large model systems over long-term simulations, while static high-level DFT calculations allow the precise calculation of geometrical and energetic parameters for small models. Gas phase high-level DFT calculations were performed in order to characterize the properties of isolated molecules and their coordination complexes with Na^+^ cations, as discussed in Section 2.1 and Section 2.3, respectively. In Section 2.2, classical MD simulations are used to describe their behavior using an extended model of the Na-Mt basal surface in the presence of water and Na^+^ cations. Finally, in Section 2.4, classical MD simulations are run in order to describe the geometrical molecular arrangement in the interlayer space of Na-Mt.

### 2.1. DIA and OXA Geometrical Characteristics

**Figure 2:**
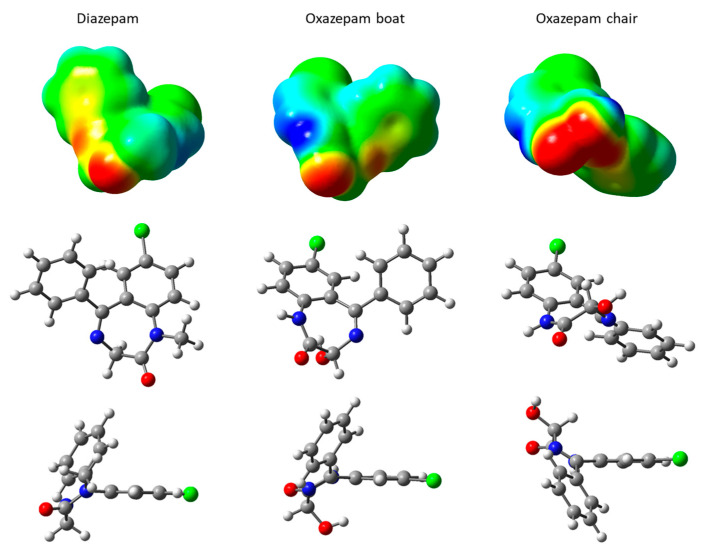
Structures and molecular electrostatic potential, mapped on an isodensity surface of DIA and OXA (R) molecules. MEP map scale: red regions correspond to negative values of the electrostatic potential, while blue equate to positive values; green regions correspond to values near zero. Atom color codes: H, white; C, gray; N, blue; O, red; Cl, green.

DIA and OXA molecules are quite similar, with a seven-membered ring comprising two N heteroatoms and carbonyl oxygen, bridged by a chlorobenzene moiety. In the case of the OXA molecule, because of the presence of a hydroxyl, the C atom in the alpha position with the carbonyl is asymmetric, leading to R and S diastereoisomers, which are both present in a racemic mixture in the commercialized forms, as used in the cited experiments [16]. These two enantiomers do not lead to important geometric differences. However, OXA may adopt two configurations through a rotation along the N-C(OH)-C(=O)-N(H) dihedral angle, as shown in Figure 2 for the R diastereoisomer. These two forms, although not corresponding to the correct nomenclature of the standard cyclohexane conformations, will hereafter be called boat and chair conformations, according to the hydroxyl orientations lying below the seven-membered cycle or oriented almost parallel to the carbonyl oxygen, respectively (see Figure 2 for the lateral views). It should be noted that these two conformations are observed for the R or S enantiomers. The transition from the boat to the chair conformation has been computed at the DFT level and shows a rather important boat–chair electronic energy barrier, rising to 9.5 kcal·mol^−1^ and to 15.8 kcal·mol^−1^ in the reverse chair–boat direction (see Figures S1 and S2 in the Supplementary Materials). As such, the two forms were considered in the classical MD calculation in order to explore their behavior on hydrated external Na-Mt surfaces. From the molecular electrostatic potential (MEP) map (Figure 2), we can see that the red regions, corresponding to the negative values of the MEP (i.e., high electronic density values) for the DIA and OXA boat are located around the carbonyl oxygen lone pairs. A slightly negative MEP value region can also be observed around the N heteroatom for these two molecules. For the OXA chair, because of the vicinity of the N heteroatom and of the hydroxyl and carbonyl oxygen lone pairs, the region of negative MEP values is much more extended, suggesting a more important Lewis basicity for this OXA chair form.

### 2.2. Adsorption of DIA and OXA on the External Surfaces of Na-Mt

**Figure 3:**
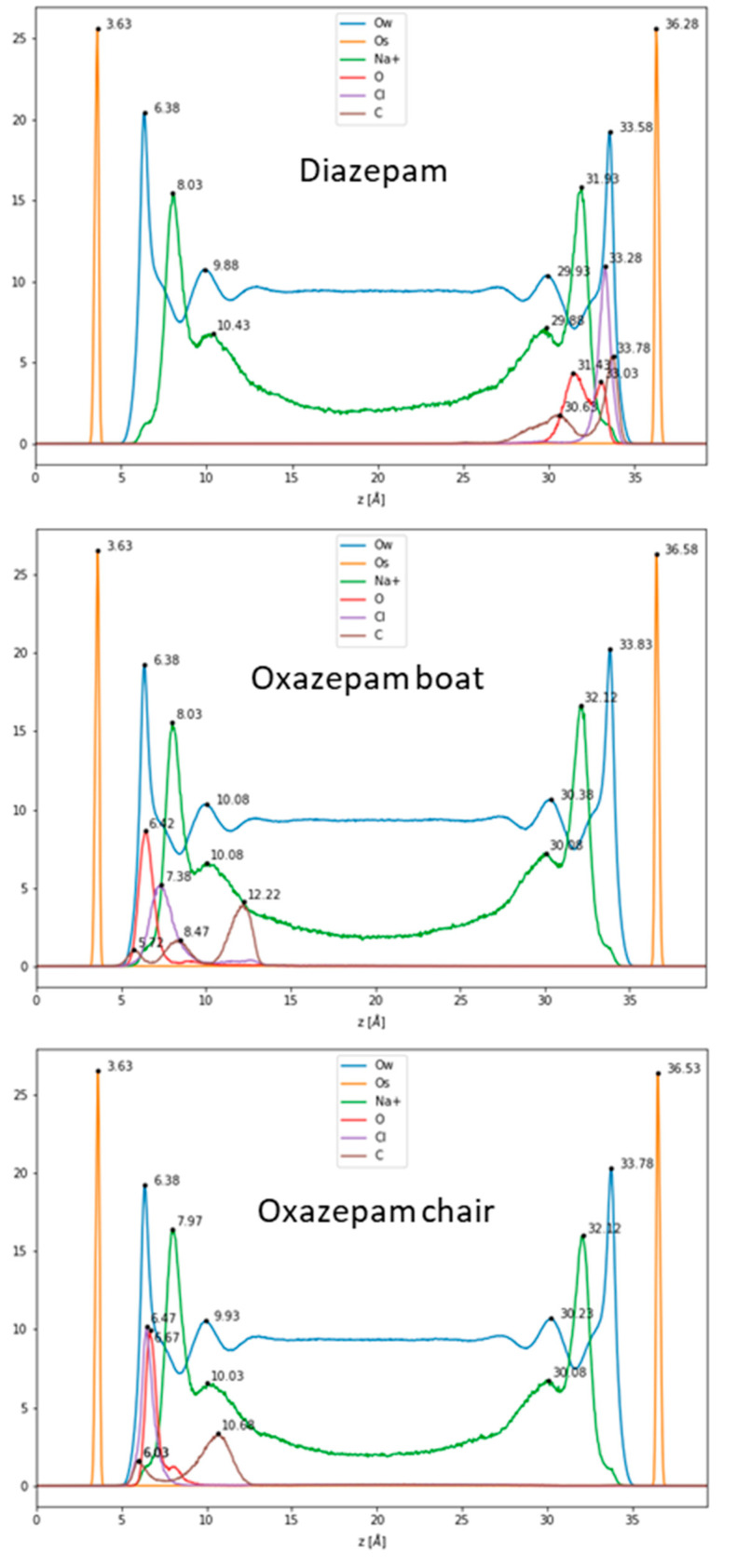
Density profiles along the orthogonal separation between the clay’s surface in the *z*-direction.

**Figure 4:**
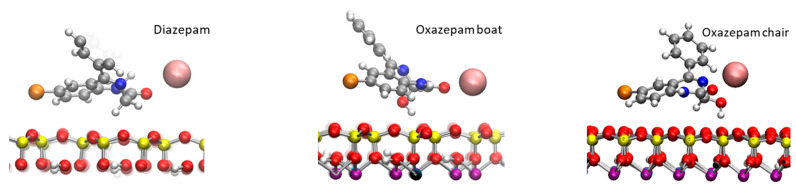
Adsorption conformations of the DIA and OXA molecules observed during the 100 ns classical MD calculations. Atom color codes: H, white; C, gray; N, blue; O, red; Cl, orange; Na, pink; Al, purple; Si, yellow.

First, 100 ns classical MDs were run for DIA, OXA boat, and chair forms, initially placed a at the center of the hydrated box between the Na-Mt external surfaces (see Figure S3 in the Supplementary Materials). Atom densities along the z direction (vertical separation between the top and bottom surfaces) are shown in Figure 3 for each molecule. The water molecules appeared to be structured in one layer at about 2.70–2.75 Å from the surface oxygen atoms, followed by a minimum density, where the cations were spread out, and a lower layer at 6.25–6.45 Å. The constant water density, beginning at around 12 Å from the surface oxygen atoms, corresponds to bulk water. The Na^+^ cations were arranged in one layer at around 4.34–4.46 Å, followed by a small shoulder 1.5–2.0 Å further on. This typical structure for external surfaces of charged clays is due to the arrangement of water molecules involved in H bonding, with negatively charged surface oxygen atoms also coordinating the mobile Na^+^ cations, compensating for the surface charges [26,33]. The density of the DIA shows a unique peak for the Cl atom at 3 Å from the surface oxygen atoms. Two peaks can be observed for the benzene C atom in the para position, centered at 2.50 and 5.65 Å from the surface, and for the carbonyl O (3.25 and 4.85 Å). These two peaks for each atom correspond to at least two adsorption modes; in one, the molecule remains planar to the surface, and in the other one, the benzene is located farther from the surface. It should be noted that in the planar configuration, the DIA is positioned fairly close (2.50–3.25 Å) to the surface, being stabilized through van der Waals interactions with the surface via its aromatic rings. For the OXA boat molecule, single peaks can be observed for the carbonyl O and Cl atoms at 2.79 and 3.75 Å, respectively. The benzene moiety appears to be more mobile through these three peaks, the furthest of which (para C, standing at 8.59 Å from the surface) corresponds to a more or less orthogonal configuration with respect to the surface. A small peak, close to the surface (2.09 Å), and a second (4.84 Å) show that a planar adsorption conformation may also, somehow, be less frequently observed. For the OXA chair, thin single peaks for the carbonyl O and Cl atoms are found at 2.84 and 3.04 Å from the surface oxygen atom, a distance comparable to water molecules’ oxygen atoms. The benzene moiety also appears to be fairly mobile and may adopt an orthogonal or planar conformation with the surface, as observed for the OXA boat molecule. The observed representative adsorption modes are shown in Figure 4. 

The radial distribution functions (RDF) of Na^+^ cations were computed around the Lewis basic atoms of the OXA and DIA molecules, i.e., the carbonyl O, the N heteroatom, and the hydroxyl O. The data are summarized in Table 1 (graphs can be found in Figure S4 in the Supplementary Materials). For the DIA and OXA boat, Na^+^ was coordinated through the carbonyl O at a distance of 2.41–2.43 Å, with coordination numbers (CN) of 0.54 and 0.32, respectively. The N heteroatom coordination cannot be considered effective since the distance values are about 4.42–4.46 Å and, thus, correspond to the coordination of the carbonyl O. This shows that the coordination DIA and OXA boat are present but are relatively moderate. The coordination to the hydroxyl O is negligible in the case of the OXA boat. For the OXA chair, the values are different and do correspond to a strong double coordination with both the carbonyl and hydroxyl oxygen atoms at 2.44–2.46 Å, with a coordination number larger than 1, thus corresponding to at least one coordinated Na^+^ atom during the whole simulation. This coordination through the N heteroatom, thereby corresponding to a triple coordination, is also observed (at 2.85 Å) at a lesser rate (CN = 0.35). These data show that because of the vicinity of Lewis basic atoms in the chair conformation, OXA behaves differently from the OXA boat or DIA near the Na-Mt surface, where the Na^+^ atoms are located. This justifies the thin peak observed for the carbonyl O in the density profile that was observed for the OXA chair. In addition, the RDF of surface oxygen atoms around the hydroxyl H of OXA has been computed (Table 1). The results show that hydroxyl is involved in H bonding with surface oxygen quite strongly in the case of the OXA chair, compared with the OXA boat (1.39–1.41 Å), with coordination numbers of 0.98 and 0.68, respectively. In the chair conformation, OXA is thus engaged in a double coordination with the Na^+^ cations near the surface and in H bonding with the surface. Van der Waals interactions with the benzene ring also stabilize both the DIA and OXA molecules in an almost planar conformation on the surface. The stronger Na^+^ coordination to one conformation of OXA, as observed in these classical MD simulations, relies on fixed parametrized charges. It is, thus, important to confirm these observations using high-level calculations.

### 2.3. Gas Phase Na^+^ Coordination Interactions Derived from DFT Calculations

**Figure 5:**
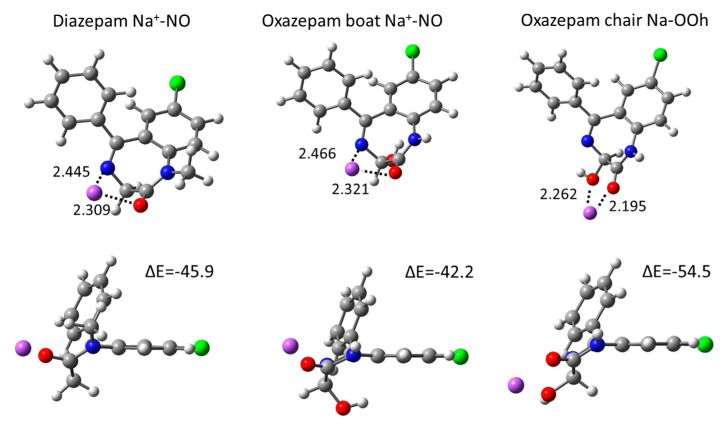
The most stable DIA and OXA coordination structures computed in the gas phase from the DFT calculations. Distances are shown in Å, electronic interaction energies in kcal·mol^-1^. Atom color codes: H, white; C, gray; N, blue; O, red; Cl, green.

All possible coordination conformations were computed in the gas phase at the DFT level, in order to evaluate accurately the interaction of DIA and OXA with one Na^+^ cation. A summary of the energetics and geometrical data are shown in Table 2 and selected conformations in Figure 5 (all computed coordination conformations can be found in Figures S5–S7 in the Supplementary Materials). For DIA and the OXA boat, the most stable conformations are found via the coordination of both the carbonyl O and the N heteroatom, with interaction energies of −45.9 and −42.2 kcal·mol^−1^, respectively. The coordination values via only the carbonyl O, which correspond to those observed from the classical MD simulations, are rather close in energies, at −42.2 and −41.3 kcal·mol^−1^ for DIA and the OXA boat, respectively (see Table 2). For these conformations, the Na-O distances are about 2.1 Å, being somewhat smaller than the ~2.4 Å distances found for the classical MD RDF values, which is due to the presence of water coordinating Na^+^. For the OXA chair, the coordination interaction values are about 10 kcal·mol^−1^ greater compared to those for DIA and the OXA boat, with the largest interactions being observed from double coordination via the carbonyl and hydroxyl oxygen atoms (ΔE = −54.5 kcal·mol^−1^). In all these OXA chair conformations, the hydroxyl oxygen atom is involved in Na^+^ coordination; thus, this confirms that the presence of Oh hydroxyl oxygen atom in the vicinity of O in the OXA chair leads to a stronger coordination with Na^+^.

### 2.4. Intercalation of OXA Chair

Taken together, the results of the 100 ns classical MD and the DFT coordination conformations show that the OXA chair behaves differently from the DIA or OXA boat molecules at the Na-Mt surface because of Na^+^-coordination. Experimentally, a larger adsorption of OXA molecules was observed compared to DIA. Since these molecules are not charged at a neutral pH, adsorption does not occur through cation exchange but rather through van der Waals interactions with the surface, coupled with their coordination with Na^+^ cations present at the surface. The larger adsorption amount of the OXA molecules can, thus, be explained by a stronger coordination with the Na^+^ cation of the OXA chair conformation. In addition, using X-ray diffraction, the intercalation of only the OXA molecule was observed when its concentration in solution increased, as soon as the lowest concentration of 10 mg.L^−1^ was surpassed. This shows that intercalation occurs at low concentrations, although it does not lead to a large number of adsorbed molecules. Indeed, this does not correspond to a cation-exchanged adsorption mode, which would lead to a larger adsorption amount; rather, this completely agrees with adsorption driven by the coordination with Na^+^ cations, which is only strong enough for the OXA molecule compared to DIA (by 10 kcal·mol^−1^ larger in energy), and, more particularly, to the OXA chair conformation, enabling a double robust Na^+^ coordination.

**Figure 6:**
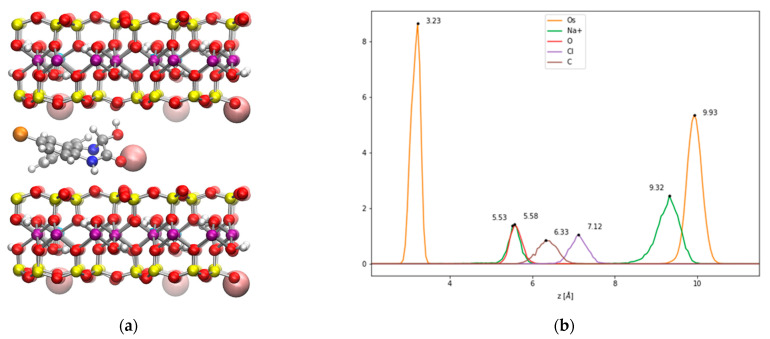
(**a**) A snapshot of the classical MD simulation of the OXA chair in the Na-Mt interlayer; (**b**) the corresponding density profile (distances are given in Å).

As such, the intercalation of the OXA chair in Na-Mt has been modeled through classical MD simulations of the interlayer Na-Mt model from dry to fully hydrated states, in order to compare the experimental d001 interlayer distances from X-ray diffraction with the distances obtained from the present simulations. The results given in Table 3 show that the value for the computed interlayer distance for dry Na-Mt was underestimated by 0.9 Å, compared to the value found in the experimental work investigating the intercalation of OXA [16]. However, our computed value is in good agreement with previous results from classical MD [34], DFT calculations [35], and XRD [36,37] experimental works, which reported values of 9.52, 9.47, and 9.60 Å, respectively. In the case of the OXA chair intercalated system without water molecules (Figure 6a), the d001 is evaluated at 13.1 Å, which is in very good agreement with the experimental value of 13.3 Å. The density profile corresponding to 1 ns of NPT classical MD is given in Figure 6b. Since this is an NPT simulation, the *c* parameter of the box (related to the vertical separation between layers) fluctuates during the simulation, leading to the atom’s peaks being a little broader than those for a fixed-dimension box. It can be seen that three Na^+^ cations remain close to the surface substitutions, while one Na^+^ is coordinated through the carbonyl and hydroxyl oxygen atoms. The RDF computed for these atoms (see Figure S8 in the Supplementary Materials) show average coordination distances of 2.25 and 2.39 Å for the carbonyl and hydroxyl oxygen atoms, while the N heteroatom remains further away at 4.12 Å. As observed in the simulation of the external surfaces, the OXA chair hydroxyl is involved in H bonding with the surface oxygen atoms, at an average distance of 1.31 Å, thereby showing an important H-bonding interaction. The OXA chair molecule remains in the center of the interlayer, thereby maximizing the van der Waals interactions with both surfaces. These observations show that this intercalation is favored by important stabilizing molecular interactions.

In order to explore whether some water molecules would change the interlayer distance, in case some molecules might have remained intercalated during the experimental drying process, we investigated a similar simulation using increasing numbers of water molecules, from 5 to 35, in the interlayer space. The results in Table 3 show that d001 tends to decrease in the presence of a few water molecules, directly coordinating the cations until 10 water molecules are reached, thus decreasing the cation’s negatively charged stabilizing interactions. Then, after 15 water molecules, the interlayer distance increases, and the layer of water molecules starts to broaden until two water layers can be observed (all data can be found in Figures S8–S15 in the Supplementary Materials).

## 3. Conclusions

The contrasting adsorption process for Na-Mt, as observed experimentally [16] for DIA and OXA comparable molecules, has been analyzed through atomistic simulations, allowing us to understand the reasons for the observed intercalation for only OXA. First characterizing the structural differences between each molecule through DFT calculations, beyond the presence/absence of methyl and hydroxyl groups, we observed that OXA can be found in two different boat and chair conformations, bringing closer the hydroxyl and carbonyl groups in the latter form. Classical MD simulations involving the external surface of Na-Mt indicated the better Na^+^ coordination of the OXA chair through a double coordination mode, compared to the OXA boat and DIA molecules. The high-level DFT computed interaction energies showed that the OXA chair does, indeed, allow for better stabilization of the doubly coordinated complex, by almost 10 kcal·mol^−1^. Since XRD experimental measurements have been performed at a neutral pH, a neutral molecule could not be involved in cation exchange. However, our calculations do reveal that, in particular, OXA chair intercalation does occur because of a strong coordination with Na^+^ cations. As such, an intercalated OXA chair molecule in a dry Na-Mt has been modeled through classical MD simulations, showing an interlayer distance that is in very good agreement with the distance that was established experimentally. In conclusion, the difference in the adsorption process observed experimentally is, thus, due to a selective intercalation between the DIA and OXA molecules, and, in particular, because of the strong Na^+^ coordination with only the chair form of OXA.

The surprising results detailed in the present study allow us to highlight the different adsorption mechanisms of similar molecules on clay minerals and, thus, their selective adsorption. This might lead to very interesting applications in the removal of pollutants from wastewater containing the target organics according to the molecules’ geometrical properties, as well as the clays’ chemical compositions. It also demonstrates the rich complementarity between experimental and theoretical approaches.

## 4. Computational Methods

### 4.1. Classical MD Simulations on the Basal Surface

Clay models were built from the X-ray data of pyrophyllite [38] and further refined through DFT calculations. Unit-cell dimensions and atom positions were optimized through periodic plane wave calculations with the VASP package [39,40], using the PBE function. The optimized cell parameters show only small variations compared to the experimental parameters (less than 2% variation): *a* = 5.083 Å, *b* = 8.897 Å, *c* = 9.410 Å, α = 90.12°, β = 100.58°, γ = 89.94°. In order to model the basal surface, only one layer was considered and the cell was changed to an orthorhombic form by multiplying *a* × 6 and *b* × 4, leading to box basal dimensions of *a* = 30.498 Å and *b* = 35.588 Å. Clay composition was set according to the better fit used in experiments [Al_3.01_Fe^(III)^_0.41_Mn_0.01_Mg_0.54_Ti_0.02_] [Si_7,98_Al_0.02_] O_20_(OH)_4_ [16]. Na-Mt was, thus, modeled by substituting 12 Al^3+^ with Mg^2+^ and 10 Al^3+^ with Fe^3+^ in the octahedral layer, leading to a unit cell composition of [Al_3.08_Fe^(III)^_0.42_Mg_0.50_] [Si_8_] O_20_(OH)_4_ Na^+^_12_. Atomic substitutions were performed randomly, respecting the rule separation between octahedral substitutions, with at least one octahedral center between two substitutions. Na+ counter-cations were included in the solution according to the number of substitutions in the mineral layer, leading to a neutral simulation box.

The ClayFF force field was used for the clay models [41], while water and ions were modeled using the SPC/E and Dang models, respectively [42,43]. The SPC/E model is known for accurately reproducing water’s structure. The DIA and OXA geometries and charge parameters were refined through DFT calculations at the B3LYP/6-31G* level, using the Gaussian package [44]. The molecules were optimized in the gas phase, and then RESP atomic partial charges were used to reproduce the electrostatic potential on a molecular surface [45]. A general AMBER force field [46] was used to generate the DIA and OXA parameters and Lorentz–Berthelot rules were applied for inter-force-field parameters. This approach has already been used successfully in various theoretical works [47,48,49,50]. The AMBER force field concept has been used extensively in nucleic acid modeling and reproduces well the structure and dynamics of nucleic acid moieties [26,33,47,48,51]. AMBER and ClayFF are quite simple to combine, since both use harmonic potential for bond terms, although ClayFF uses neither angle nor dihedral terms. Periodic boundary conditions were applied in all three spatial directions.

The large-scale atomistic/molecular massively parallel simulator (LAMMPS) [52] was used for all simulations. A unique clay layer was considered for modeling the basal surface and this was divided, half at the bottom and half at the top of the box, along the z vertical parameter (see Figure S1 in the Supplementary Materials). The initial separation was set to 60 Å, the DIA or OXA molecule was placed in the center of the box, and water molecules filled the empty space in a regular grip (with a 3.3 Å interval), including the amount of Na+ needed for a total neutral charge. A cutoff of 12 Å was applied to the electrostatic and Lennard–Jones interactions. Coulombic interactions were computed via Ewald summation and the particle–particle, particle–mesh method at an accuracy of 0.0001. All simulations were carried out at 300 K and 1 atm. The oxygen–hydrogen bonds of water molecules were maintained as fixed by applying the shake algorithm [53]. The equilibration period consisted of warming up the system, starting with the water/ions moiety, then the clay particles, and, finally, the nucleotide, via the NVT ensemble. The system was then allowed to relax in all directions through the NPT ensemble. This step allowed us to fix the vertical separation between the basal surfaces for the whole production. The system was then left to equilibrate via two steps, increasing the time step from 0.1 to 0.5 fs. The whole equilibration process took 800,000 time steps. During the whole simulation and production, the octahedral atoms were maintained as fixed. The production time during which the data were analyzed was 100 ns for all simulations.

### 4.2. Gas Phase DFT Calculations

The stable geometries of the DIA and OXA molecules were optimized through B3LYP/6-311G* calculations from the Gaussian package [44]. Complexed geometries with Na^+^ were also characterized by optimizing several possible coordination states. The coordination energies were simply computed as the difference between the energy of the complex and those of the isolated molecules and the Na^+^ cation. For OXA, the R and S enantiomers, as well as the boat and chair forms, were considered. The transition state from the boat to the chair form was also characterized using a relaxed scan of the dihedral angle involved in the conformational change, then the highest point was used for the assumed TS structures needed for a standard QST3 refinement. The obtained TS geometry was characterized using a frequency calculation, highlighting a unique negative frequency corresponding to the rotation along the dihedral angle involving the hydroxyl group, as well as the rotation of the benzene group. The boat and chair geometries were confirmed by following and optimizing structures from irc calculations (geometries and irc data are shown in Figures S1 and S2 in the Supplementary Materials).

### 4.3. Classical MD Simulations of the Interlayer Space

The intercalation of the OXA boat in the Na-Mt interlayer was characterized through 1 ns of classical MD calculations on a smaller supercell created from the initial pyrophyllite unit cell (see above), with *a* × 4 and *b* × 2 dimensions. Four Al^3+^ by Mg^2+^ substitutions and Na^+^ counter were introduced. The calculation setup followed the NVT warming step (from T = 0 to T = 300 K) for 50,000 steps with a 0.1 fs time step. The system was then equilibrated at 300 K for the same number of steps, after which the time step is then increased to 0.5 fs for 50,000 steps. The production was then run for 1 ns through an NPT simulation, allowing the system to reach equilibration in three directions. These calculations were run for various water contents from the dry to the fully hydrated interlayers in order to evaluate the d001 interlayer distance, which could be compared to experiments. The starting *c* parameter of the box was set to 15 and 18 Å for the completely dry system and those comprising the OXA chair molecule, respectively.

## Figures and Tables

**Table 1:** Radial distribution function data obtained from the 100 ns classical MDs for the DIA and OXA N heteroatom, carbonyl O, and hydroxyl Oh with Na+ cations. The first peak maximum is shown in Å; CN stands for the coordination number (the corresponding RDF graphs can be found in Figure S4 in the Supplementary Materials).

	N—Na^+^		O—Na^+^		Oh—Na^+^		Hh—Os	
	1st Peak	CN	1st Peak	CN	1st Peak	CN	1st Peak	CN
Diazepam	4.42	0.64	2.41	0.54				
Oxazepam boat	4.46	0.44	2.43	0.32	2.52	0.03	1.41	0.68
Oxazepam chair	2.81	0.35	2.44	1.25	2.46	1.07	1.39	0.98

**Table 2:** DIA and OXA coordination geometric and energetic parameters. Interaction energies are given in kcal·mol^−1^ and distances are given in Å. The coordinating atoms correspond to the carbonyl oxygen O, nitrogen heteroatom N, and hydroxyl oxygen Oh.

	Coordinating Atoms	ΔE	d Na—O	d Na—N	d Na—Oh
Diazepam	O	−42.2	2.100		
	N	−41.4		2.311	
	O N	−45.9	2.309	2.445	
Oxazepam boat	O	−41.3	2.102		
	N	−39.6		2.317	
	O N	−42.2	2.321	2.466	
	N Oh	−34.0		2.477	2.311
	Oh π	−36.7			2.240
Oxazepam chair	O Oh	−54.5	2.195		2.262
	N Oh	−52.0		2.331	2.279
	N O Oh	−51.3	2.379	2.647	2.282

**Table 3:** The d001 interlayer distances (in Å) obtained from classical MD simulations of the Na-Mt interlayer, comprising the OXA chair molecule and an increasing number of water molecules, compared to the experimental values obtained via X-ray diffraction [16].

	Dry	OXA 0 w	OXA 5 w	OXA 10 w	OXA 15 w	OXA 20 w	OXA 25 w	OXA 30 w	OXA 35 w
MD	9.5	13.1	12.8	12.4	12.7	13.2	12.9	13.3	13.6
Exp.	10.4	13.3							

## Data Availability

Data are already joined in supplementary materials.

## Supplementary Materials

The supporting information, as cited in the text, can be found at: https://www.mdpi.com/article/10.3390/ijms241914781/s1.
