# Supplementary material for: Selective Adsorption of Organic Micro-Pollutants by Smectite Clays Revealed from Atomistic Simulations
Source: Int J Mol Sci. 2023 Sep 30;24(19):14781. doi: 10.3390/ijms241914781 (PMC10572936; doi:10.3390/ijms241914781)

SUPPORTING INFORMATION

Selective Adsorption of Organic Micro-Pollutants by Smectite Clays Revealed from Atomistic Simulations

Mathieu Cancade [1], Thomas Thiebault [2] and Pierre Mignon [1,*]

[1] Institut Lumière Matière, UMR 5306, Université Claude Bernard Lyon 1, CNRS, Université de Lyon, 69622 Villeurbanne, France; mathieu.cancade@ens-lyon.fr

[2] Milieux Environnementaux, Transferts et Interactions dans les Hydrosystèmes et les Sols, Sorbonne Université, CNRS, EPHE, PSL University, UMR 7619, 75005 Paris, France; thomas.thiebault@upmc.fr

* Correspondence: pierre.mignon@univ-lyon1.fr

Figure S1. Boat to chair reaction path energies and structures for OXA (R).

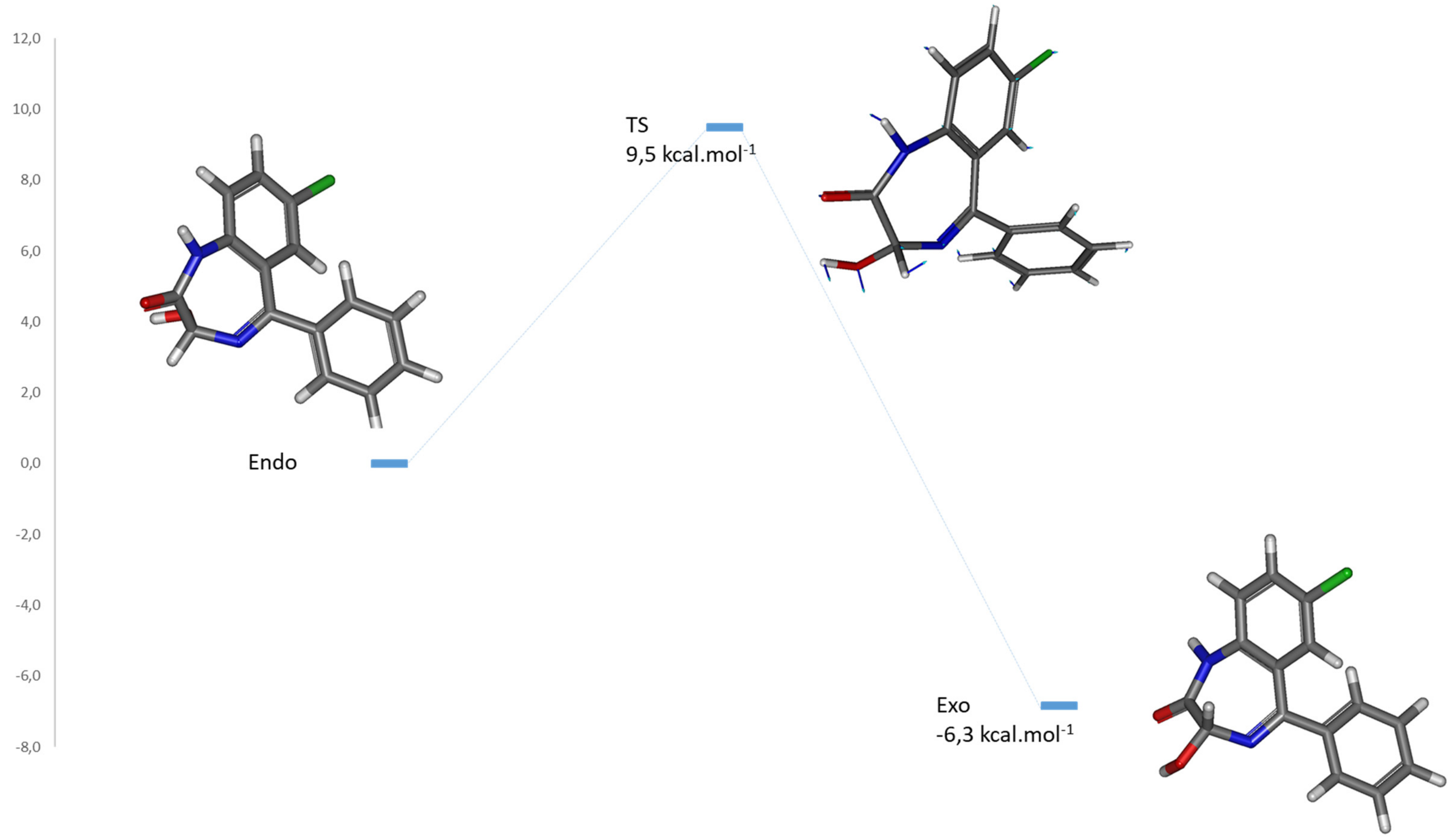

Figure S2. IRC path results

Energies reported relative to the TS energy of -1298.558462

Summary of reaction path following

	Energy	RxCoord
1	-0.00064	-6.37227
2	-0.00053	-5.61545
3	-0.00042	-4.82135
4	-0.00035	-4.16515
5	-0.00027	-3.52042
6	-0.00022	-2.98127
7	-0.00017	-2.47674
8	-0.00014	-2.00675
9	-0.00010	-1.56583
10	0.00000	0.00000
11	-0.00012	1.61580
12	-0.00016	2.11792
13	-0.00022	2.67361
14	-0.00028	3.28853
15	-0.00035	3.87257
16	-0.00044	4.61603
17	-0.00060	5.52654
18	-0.00082	6.61675
19	-0.00138	8.12604
20	-0.00176	8.86096

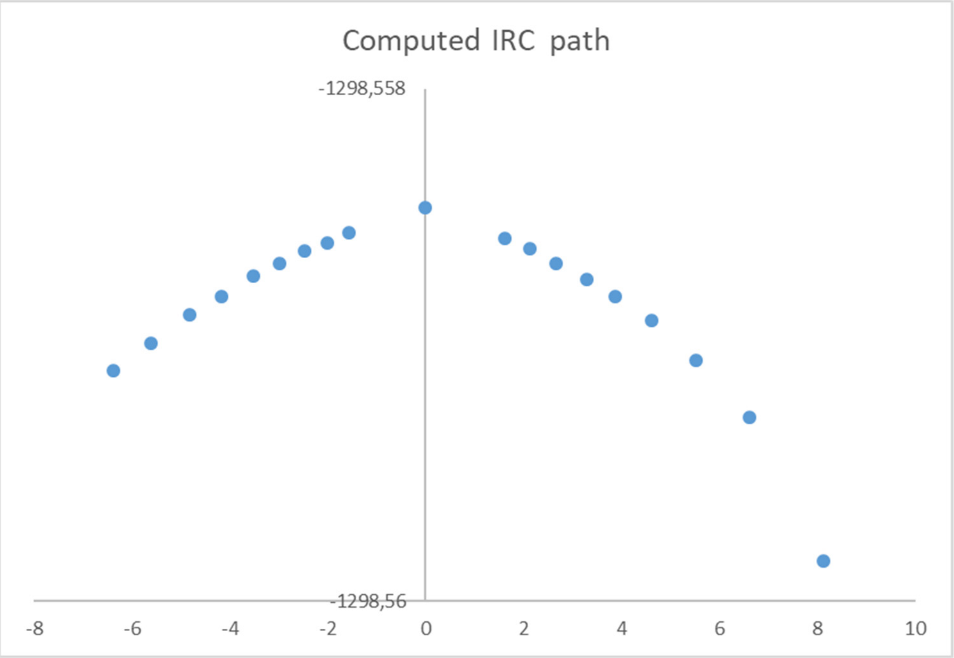

Figure S3. Snapshot of the whole simulation box with and without water for the OXA Endomolecule . The z axis corresponds to the vertical separation between the Na-Mt surfaces.

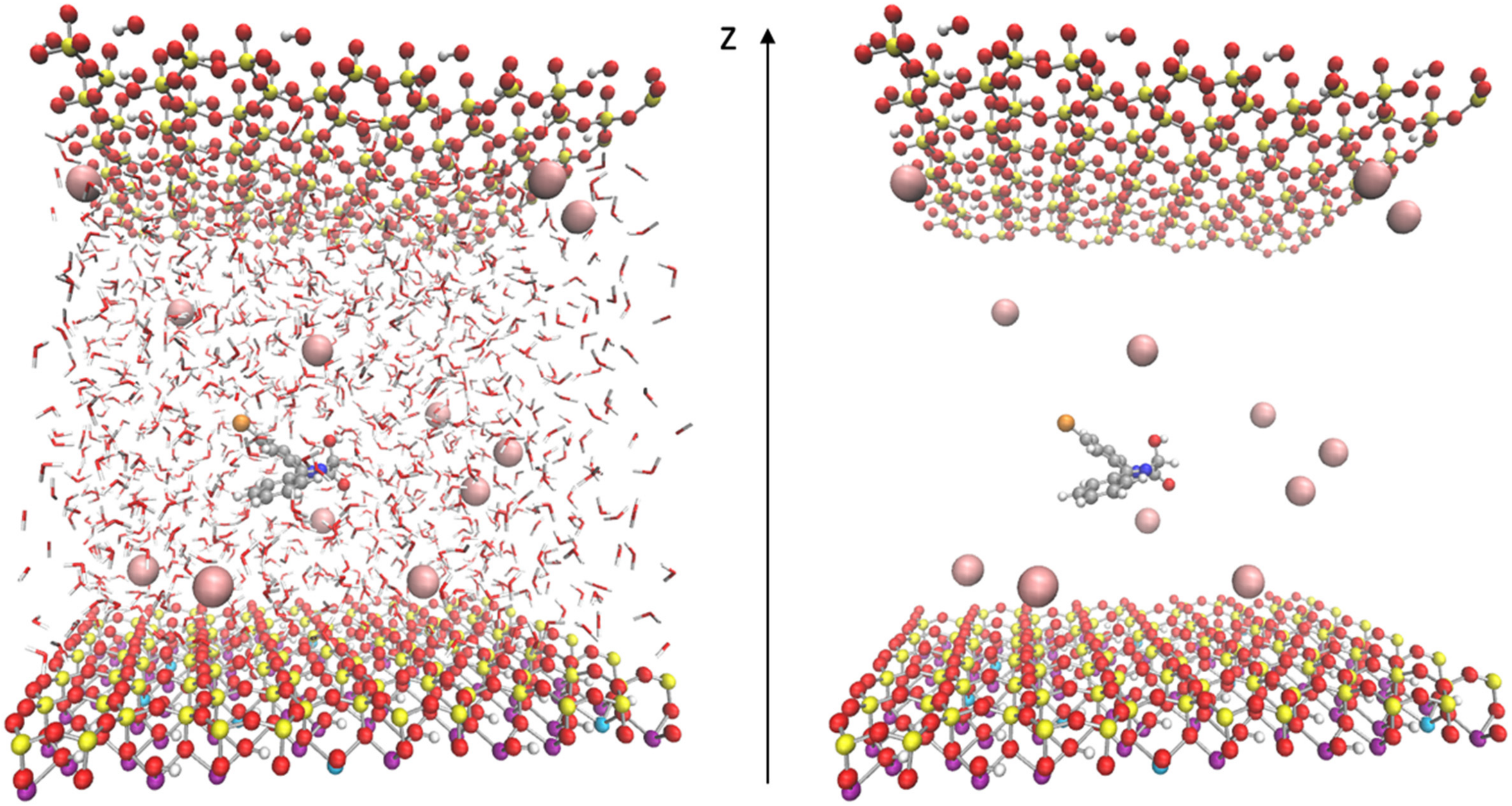

Figure S4. Radial distribution functions for DIA and OXA N heteroatom (left), carbonyl O (middle) and hydroxyl O (right) with Na^+ cations, computed during the 100ns classical MD simulations.

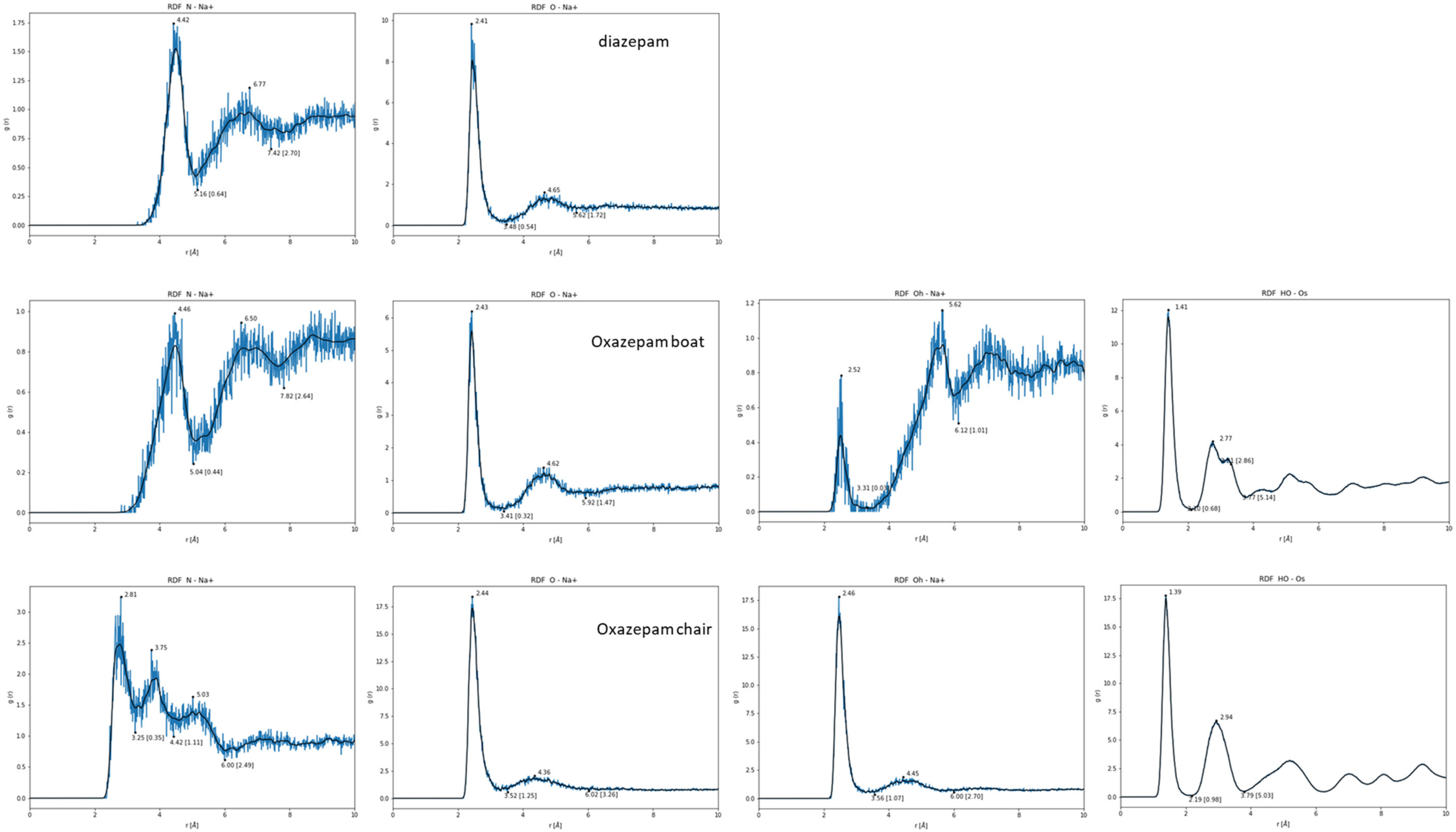

Figure S5. Geometries and interaction energies of Na^+ coordinated with DIA.

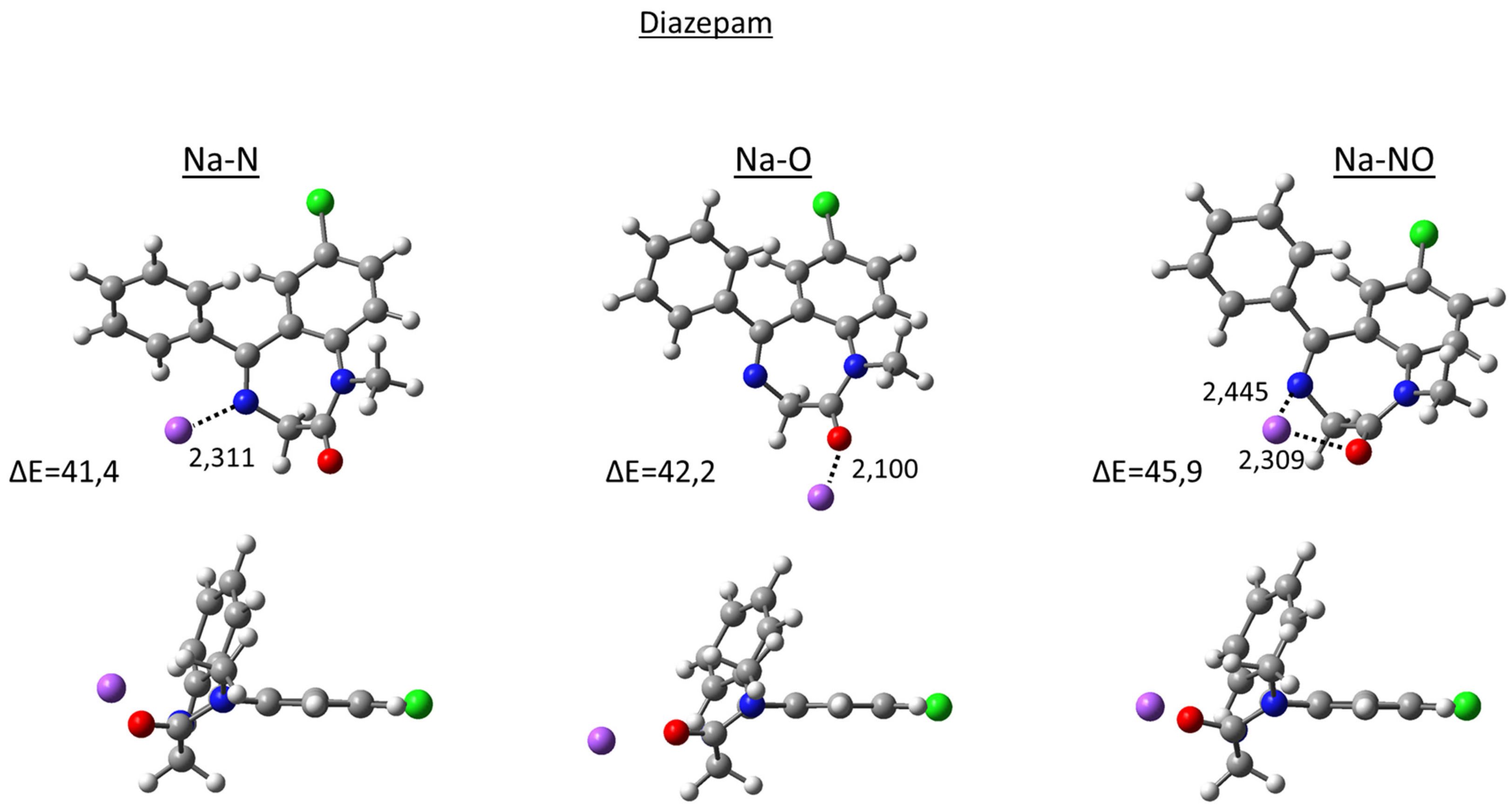

Figure S6. Geometries and interaction energies of Na^+ coordinated with OXA Endo.

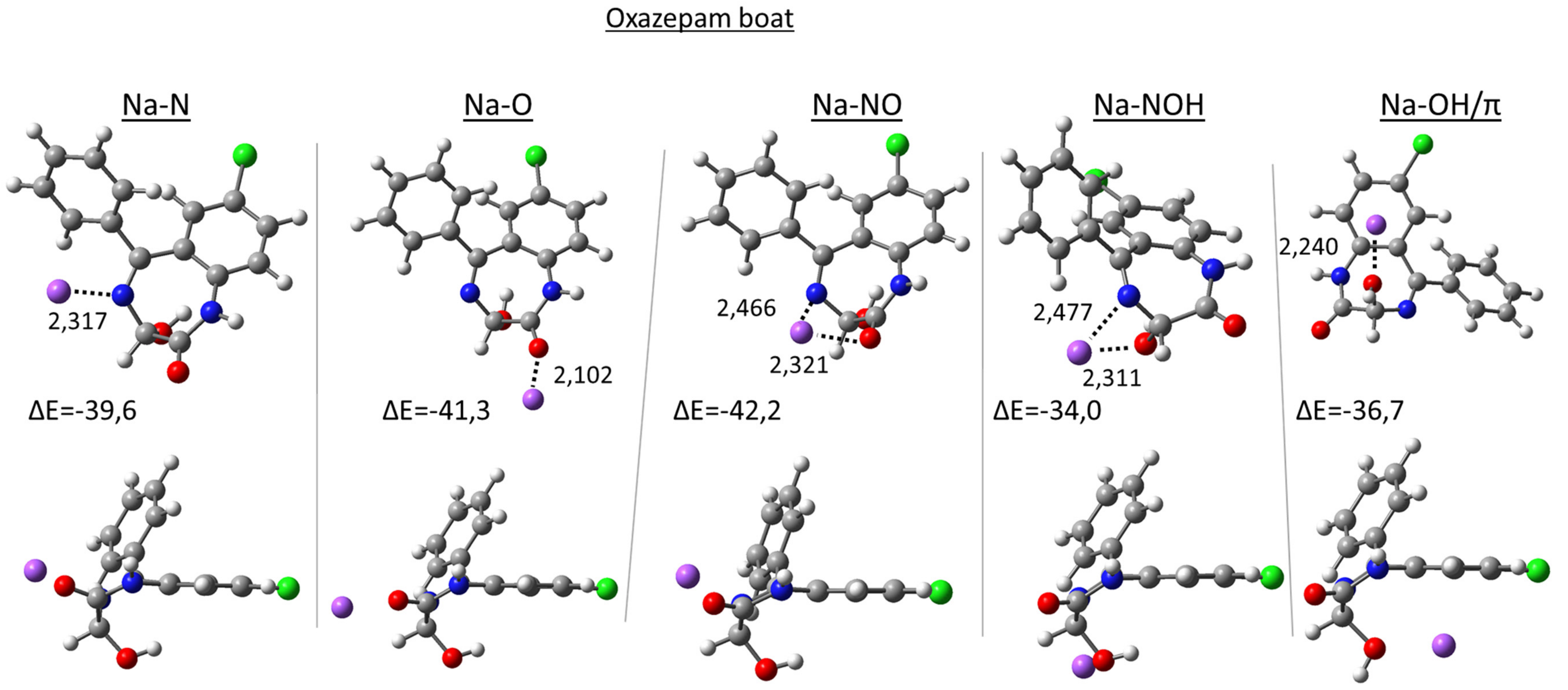

Figure S7. Geometries and interaction energies of Na^{+} coordinated with OXA Exo.

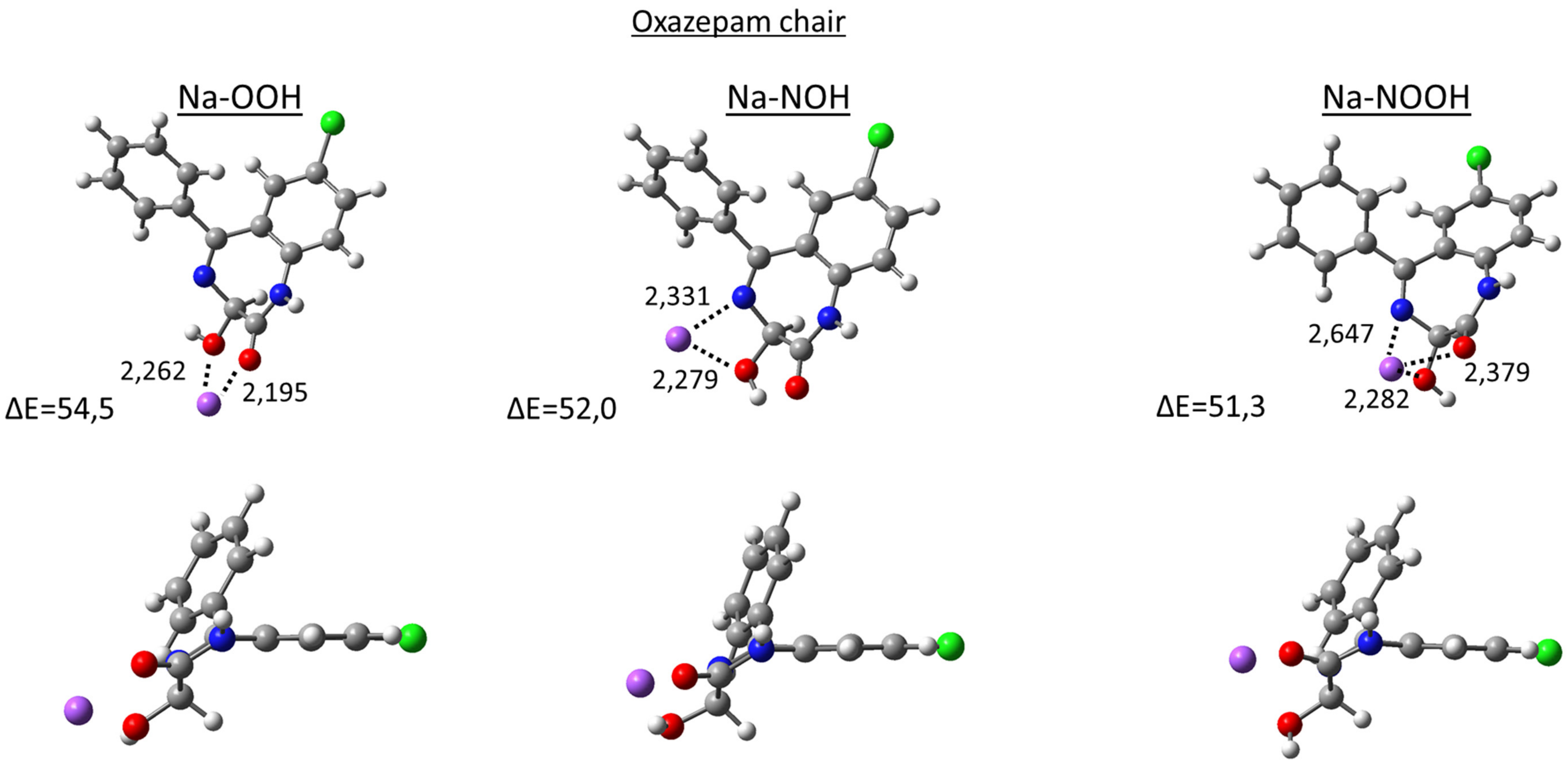

Figure S8. Simulations data for the intercalated OXA chair in the Na-Mt interlayer (No Water).

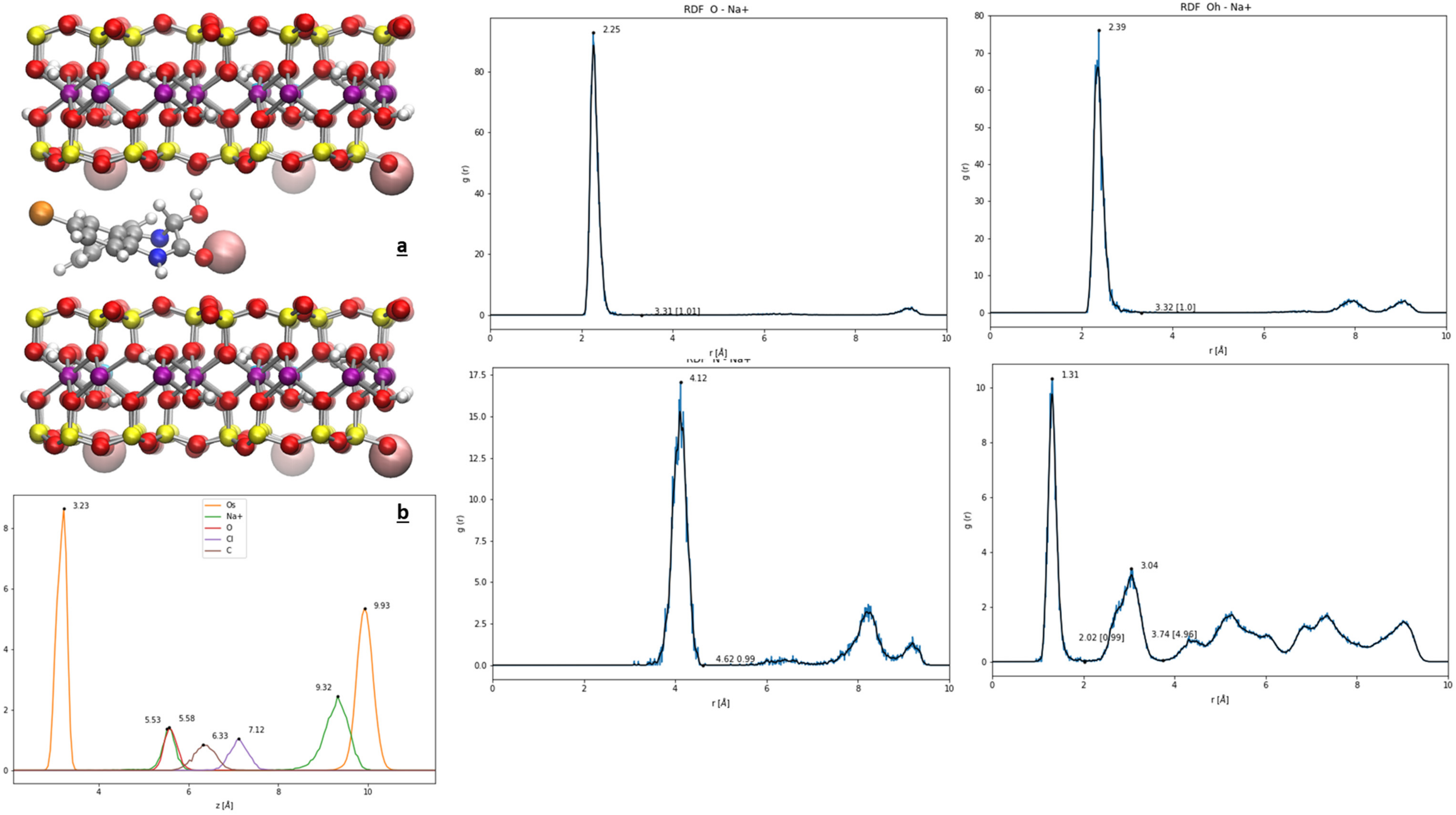

Figure S9. Simulations data for the intercalated OXA chair in the Na-Mt interlayer (5 water molecules).

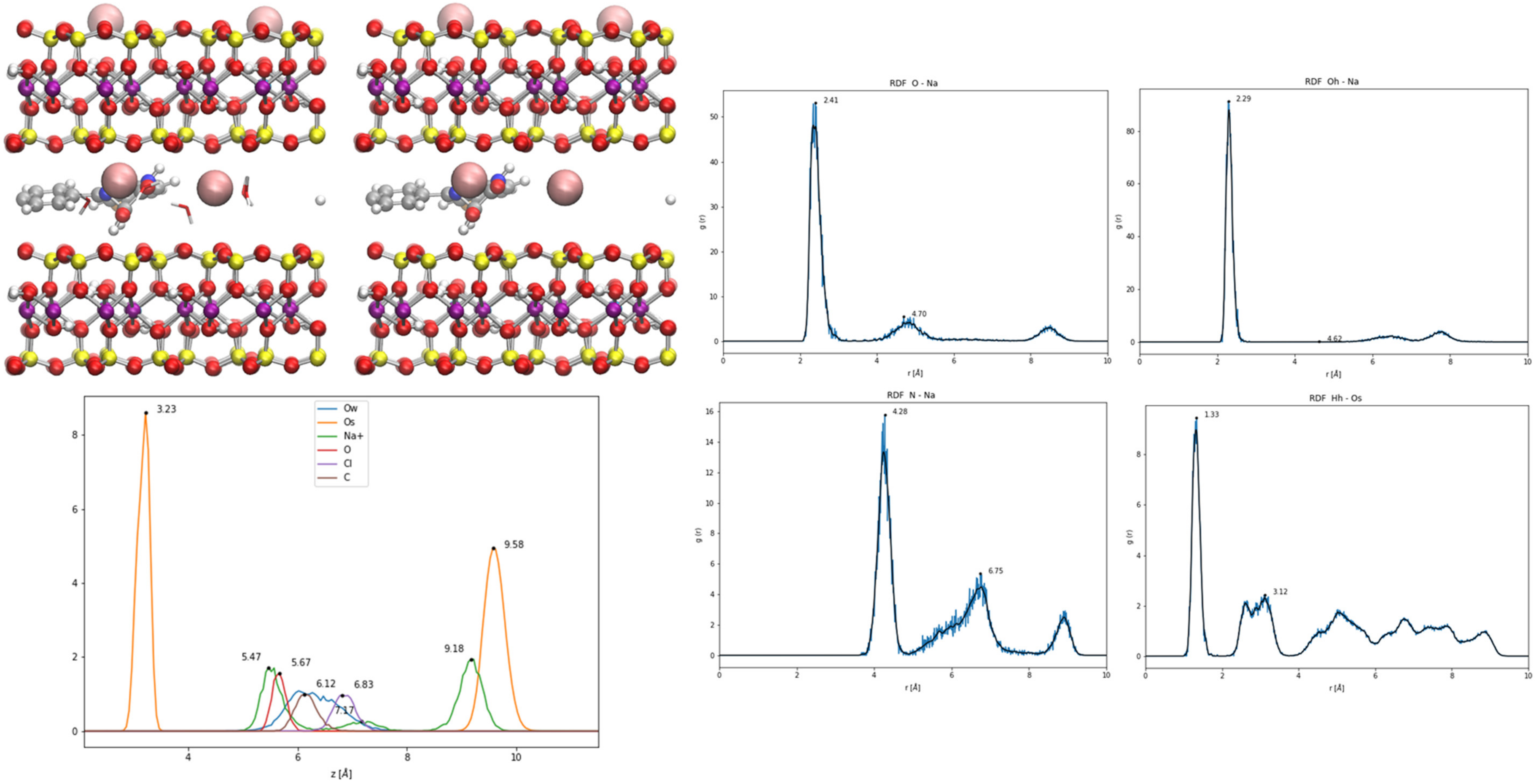

Figure S10. Simulations data for the intercalated OXA chair in the Na-Mt interlayer (10 water molecules).

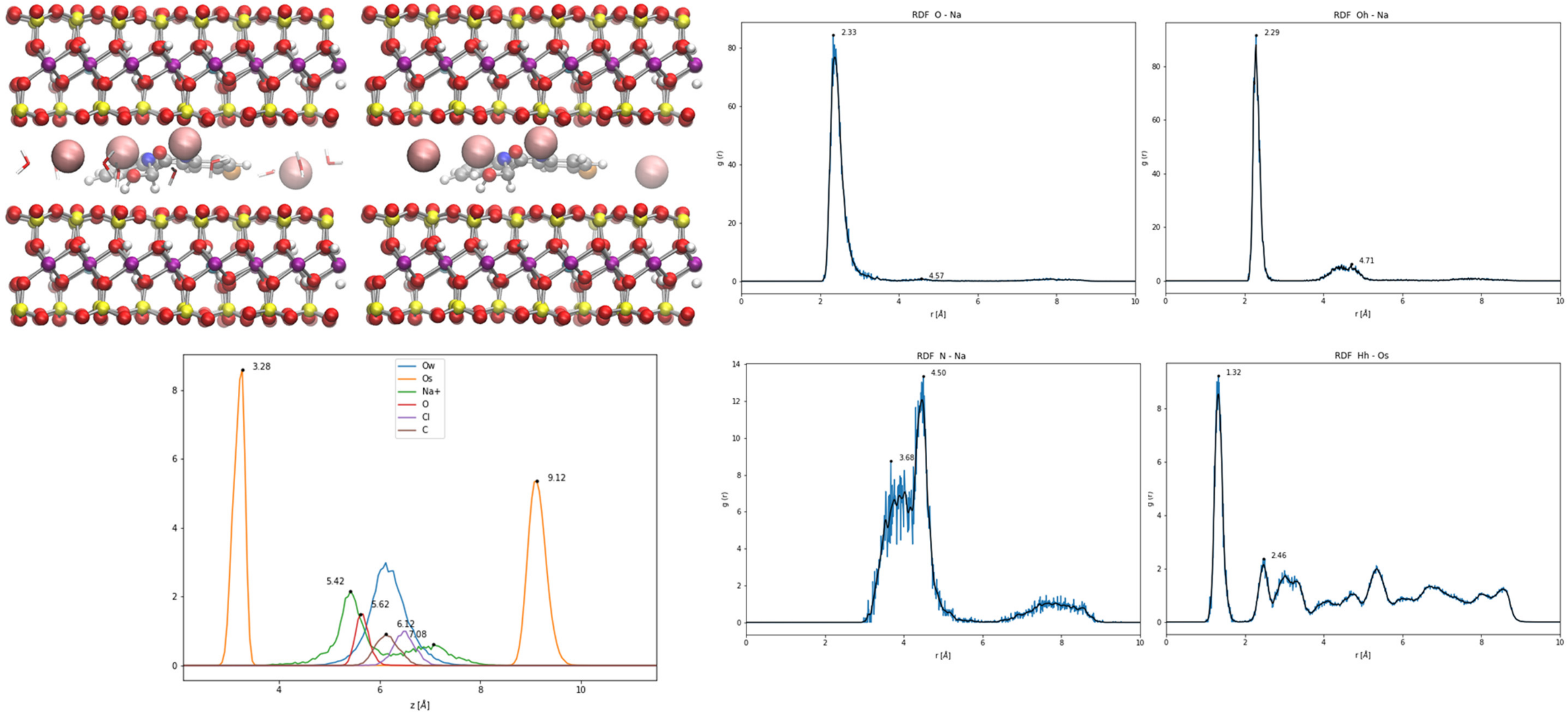

Figure S11. Simulations data for the intercalated OXA chair in the Na-Mt interlayer (15 water molecules).

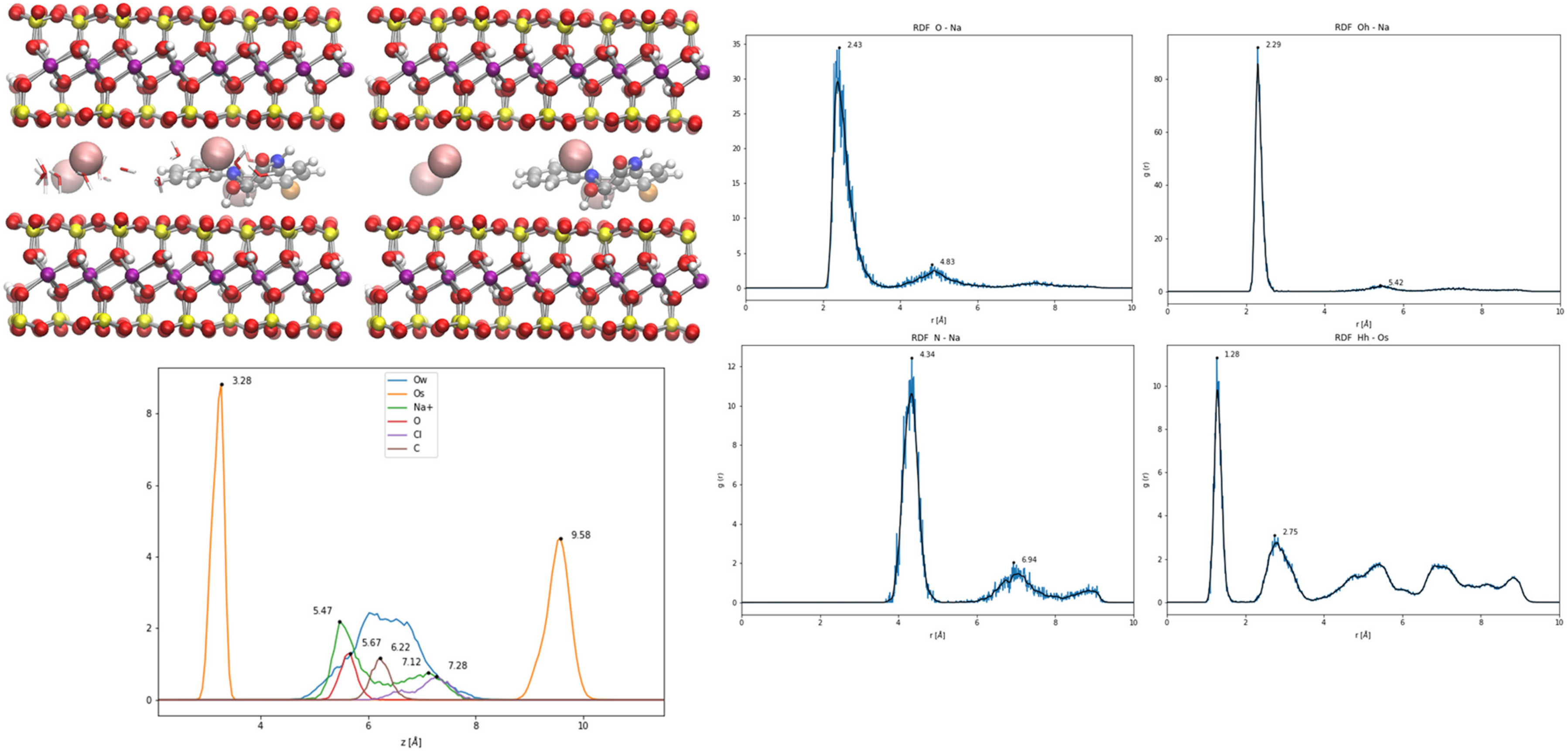

Figure S12. Simulations data for the intercalated OXA chair in the Na-Mt interlayer (20 water molecules).

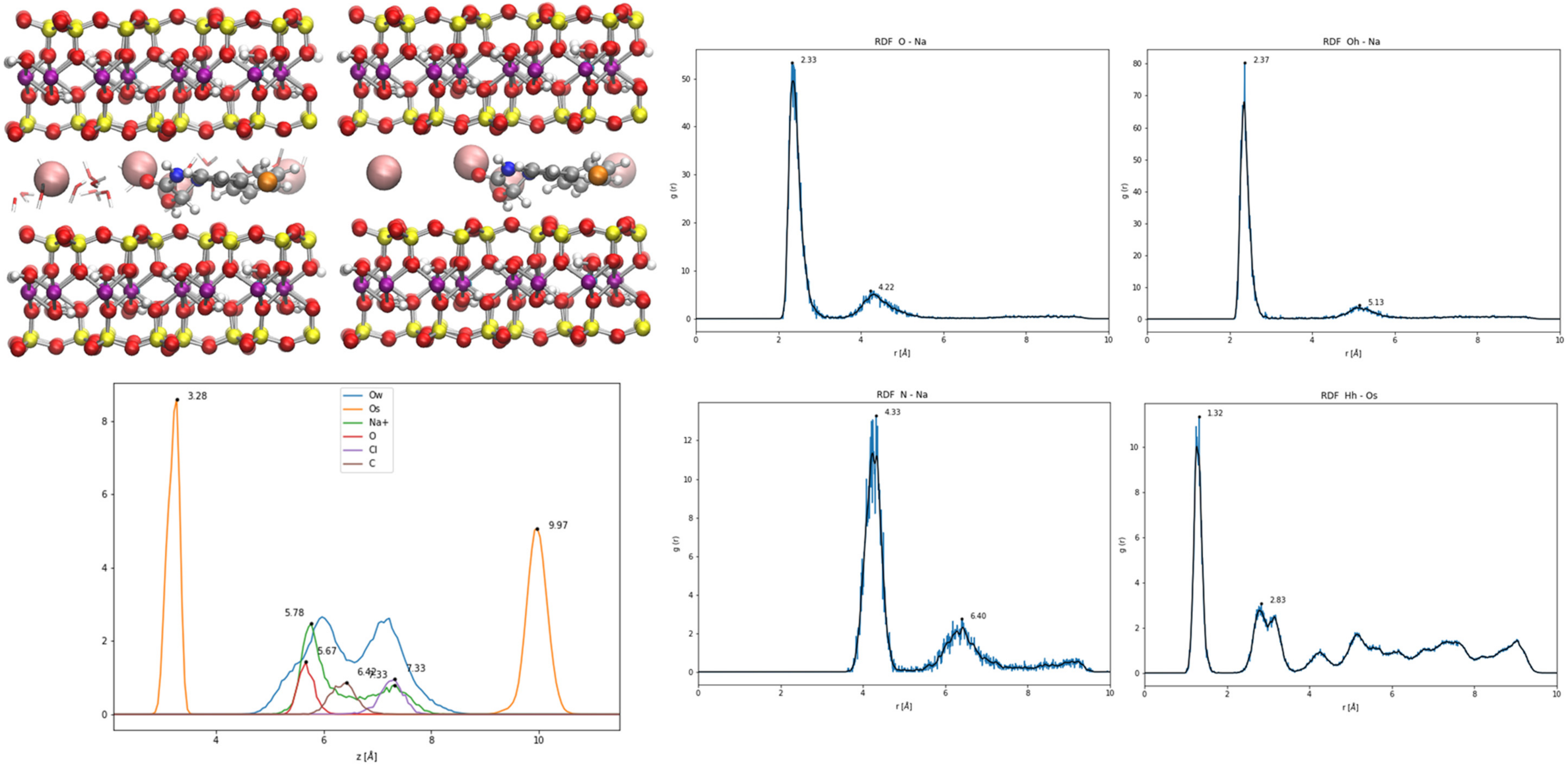

Figure S13. Simulations data for the intercalated OXA chair in the Na-Mt interlayer (25 water molecules).

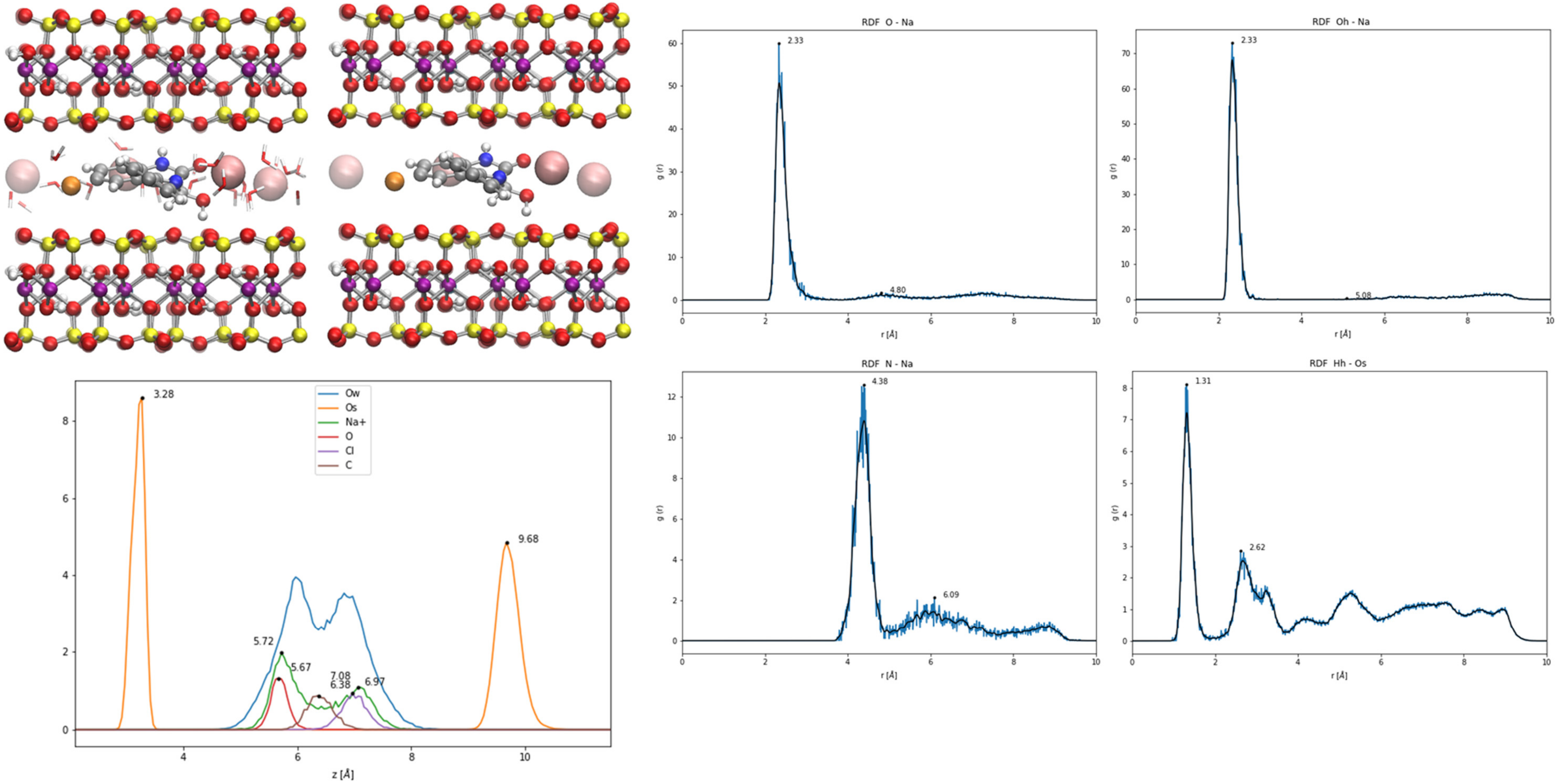

Figure S14. Simulations data for the intercalated OXA chair in the Na-Mt interlayer (30 water molecules).

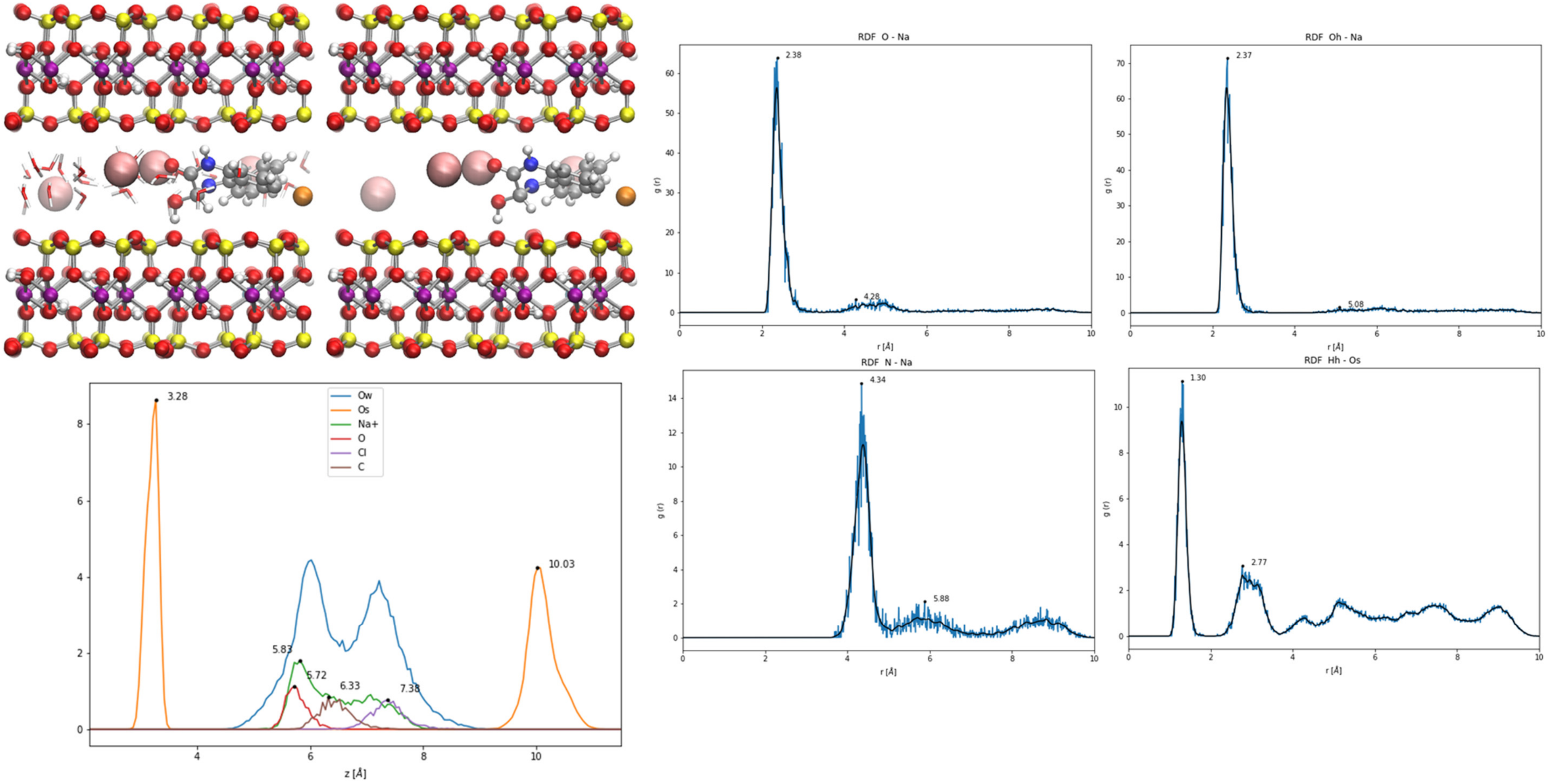

Figure S15. Simulations data for the intercalated OXA chair in the Na-Mt interlayer (35 water molecules).

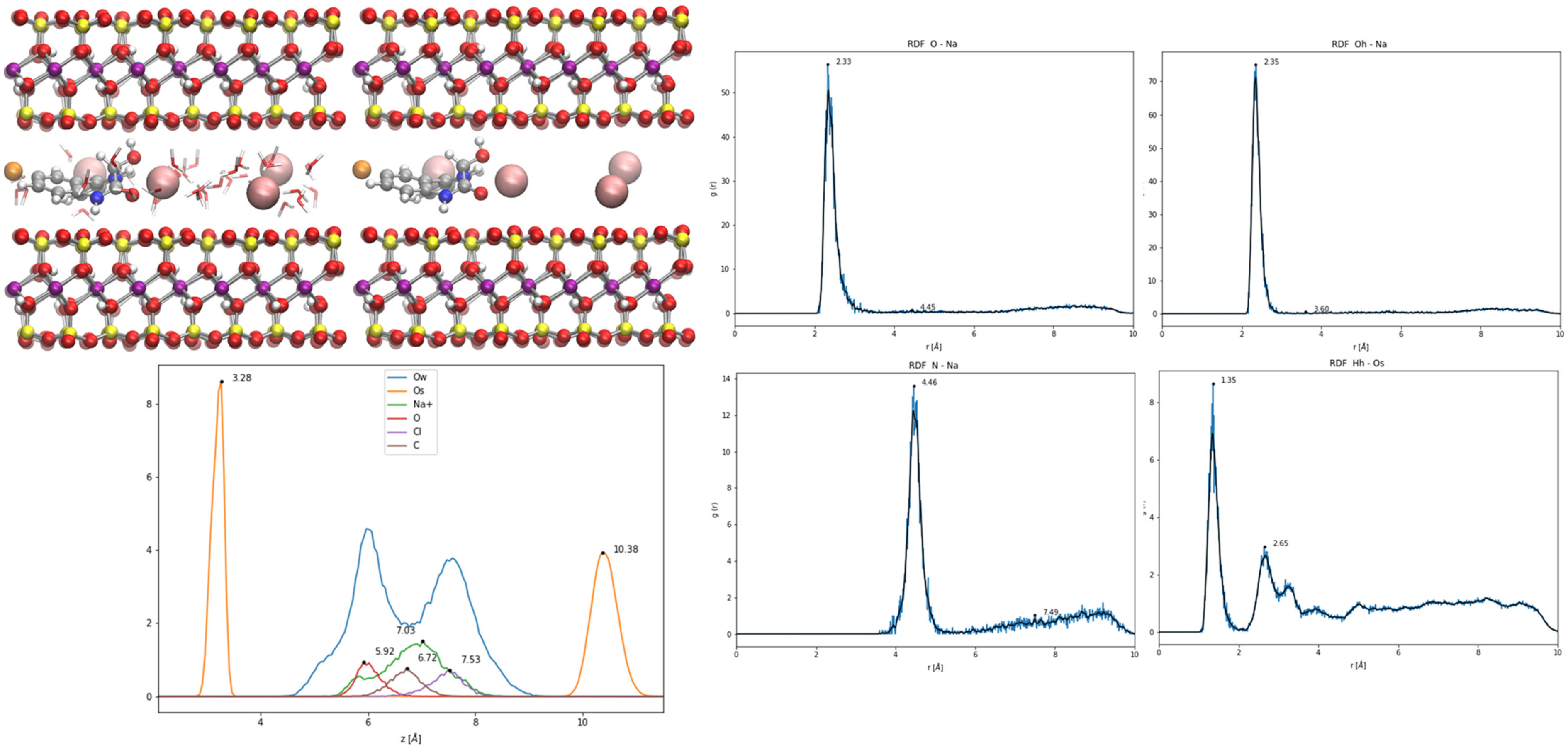

Supplement: Supplementary file 1 [file ijms-24-14781-s001.zip › ijms-2616640-SI.pdf]
